# Development and Evaluation of a Fidelity Instrument for PEARLS

**DOI:** 10.3389/fpubh.2014.00200

**Published:** 2015-04-27

**Authors:** Laura Farren, Mark Snowden, Lesley Steinman, Maria Monroe-DeVita

**Affiliations:** ^1^Health Promotion Research Center, University of Washington, Seattle, WA, USA; ^2^Department of Psychiatry and Behavioral Sciences, Harborview Medical Center, University of Washington, Seattle, WA, USA; ^3^Division of Public Behavioral Health and Justice Policy, Department of Psychiatry and Behavioral Sciences, University of Washington, Seattle, WA, USA

**Keywords:** fidelity, implementation, depression, evidence-based, older adults

## Abstract

**Purpose:**

This manuscript describes the development and the preliminary evaluation of a fidelity instrument for the Program for Encouraging Active and Rewarding Lives (PEARLS), an evidence-based depression care management (DCM) program. The objective of the study was to find an effective, practical, multidimensional approach to measure fidelity of PEARLS programs to the original, research-driven PEARLS protocol in order to inform program implementation at various settings nationwide.

**Methods:**

We conducted key informant interviews with PEARLS stakeholders, and held focus groups with former PEARLS clients, to identify core program components. These components were then ranked using a Q-sort process, and incorporated into a brief instrument. We tested the instrument at two time points with PEARLS counselors, other DCM program counselors, and non-DCM program counselors (*n* = 56) in six states. Known-groups method was used to compare findings from PEARLS programs, other DCM programs, and non-DCM programs. We asked supervisors of the counselors to complete the fidelity instrument on behalf of their counselors to affirm the validity of the results. We examined the association of PEARLS program fidelity with individual client outcomes.

**Results:**

Program for Encouraging Active and Rewarding Lives providers reported the highest fidelity scores compared to DCM program providers and non-DCM program providers. The sample size was too small to yield significant results on the comparison between counselor experience and fidelity. Scores varied between PEARLS counselors and their supervisors. PEARLS program fidelity was not significantly correlated with client outcomes, suggesting that other implementation factors may have influenced the outcomes and/or that the instrument needs refinement.

**Conclusion:**

Our findings suggest that providers may be able to use the instrument to assess PEARLS program fidelity in various settings across the country. However, more rigorous research is needed to evaluate instrument effectiveness.

## Introduction

The Program to Encourage Active and Rewarding Lives (PEARLS) was developed in the late 1990s by the University of Washington Health Promotion Research Center, in collaboration with our local Area Agency on Aging and a network of senior centers. PEARLS is a depression care management (DCM) program using the Chronic Care model (1). PEARLS includes active screening for depression, using a trained depression care manager, a team approach, stepped care, and built-in follow-up. Depression care managers deliver brief, evidence-based interventions and provide education and self-management support, proactive outcome measurement, and tracking using the nine-item Patient Health Questionnaire (PHQ-9). PEARLS is a participant-driven program, aimed at teaching individuals effective skills to manage their lives when they get overwhelmed. Case managers, nurses, social workers, and other front-line staff at community-based aging and social service agencies are trained as PEARLS counselors and work with participants, teaching them problem-solving treatment methods in combination with behavioral activation techniques.

In a randomized controlled trial (RCT), the PEARLS program was shown to significantly improve depression treatment outcomes for frail, socially isolated elders with minor depression and dysthymia when compared to the typical care that these clients received (2). Fifty-four percent of PEARLS clients showed at least a 50% decrease in the 20-item Hopkins Symptoms Checklist (HSCL-20) (3) depression score from baseline to 6-months versus 8% of clients in usual care at the same time interval. Forty-four percent of PEARLS clients showed complete remission from depression after 6-months versus 10% of clients in usual care. Compared to usual care, PEARLS participants also had greater health-related quality-of-life improvements in functional well-being (*p* = 0.001) and emotional well-being (*p* = 0.048). Thirty-four percent of individuals in usual care reported any hospitalization during the first 6 months of treatment compared to only 22% of PEARLS clients, suggesting potential health care cost savings (2). A subsequent RCT (4, 5) demonstrated the effectiveness of PEARLS in treating adults of all ages with epilepsy and major depression.

Currently, PEARLS is offered to older adults and adults with epilepsy in approximately 45 sites across 14 states. As it is disseminated nationally, and implemented in various community settings, fidelity becomes increasingly important. Implementation, as it is described in the RE-AIM framework, “refers to the extent to which a program is delivered as intended” both at the site-level and individual level (6). Fidelity is the adherence of a scientifically developed program to the original, research-based protocol, and is necessary for implementing evidence-based programs (7). The literature around evidence-based program implementation emphasizes the importance of maintaining program fidelity to ensure positive outcomes (8–10). Low program fidelity has been shown to negatively impact outcomes (8, 11). Similarly, high program fidelity has been linked to more positive outcomes across a range of evidence-based practices serving a variety of populations (12–14). Dissemination and implementation models such as the Fixsen Implementation framework (10), the RE-AIM framework (15), and the dissemination framework for evidence-based health promotion practices (16) assume that program adaptation is necessary, expected, and must inform program evolution. However, measuring program adaptations, and how those adaptations relate to client outcomes, is an essential step in determining the extent to which an evidence-based program may be modified, while continuing to remain evidence-based.

## Materials and Methods

The study was completed in three phases. The first phase involved developing a brief, multidimensional instrument for measuring PEARLS fidelity across sites. The second phase involved conducting a preliminary evaluation of that fidelity instrument. The third phase evaluated the association of PEARLS program fidelity instrument scores with individual client outcomes. This study was approved by the University of Washington Institutional Review Board.

### Instrument development

We used purposive convenience sampling to identify study participants with experience in PEARLS program development, training, delivery, and receipt. First, we recruited interview participants. The participants included program developers and researchers, program administrators, and PEARLS counselors. Using qualitative methods, we conducted 30–60 min interviews (MS, LS) with these individuals to identify the core, programmatic components of PEARLS from their perspective. Next, we recruited former PEARLS clients who had completed the program within the previous 12 months, and held focus groups with these individuals to identify the core, programmatic components of PEARLS from their perspective. We provided a $25 incentive to focus group participants. Incentives were not provided to interview participants.

After the interview and focus group participants identified program components, we used Q-sort ranking (17) to prioritize these items: we presented the list of possible items to the interview participants on a spreadsheet, along with ranking chips (four 1s, nine 2s, sixteen 3s, nine 4s, and four 5s). The interview participants ranked the items in order of priority (“5” = most core elements of PEARLS to “1” = least core elements of PEARLS), and only used the ranking chips allotted to create a normal distribution. We calculated the numbers of 1s and 2s (“low” ranking) and 4s and 5s (“high” ranking) for each of the items. We then subtracted the number of high rankings from the number of low rankings to come up with a “high-low” score for each item. Those items that received a high-low score >0 were presented back to the interview and focus group participants to confirm that no items were missing. The participants were asked to then identify any additional items. The research team then created multiple-choice questions from each of the items, with five anchor points and a scale of one to five. The interview participants also reviewed the anchor points, provided feedback, and we modified the anchor points based on that feedback. A copy of the resulting PEARLS Fidelity instrument is provided in Data Sheet 1 in Supplementary Material.

### Preliminary evaluation

We used known-groups method data analysis (18) to conduct a preliminary evaluation of the reliability and validity of the PEARLS fidelity instrument. We compared PEARLS, other DCM programs, and non-DCM programs across six states (CA, FL, GA, IL, MD, WA). The DCM programs included IMPACT (19) and Healthy IDEAS (20). IMPACT is a primary care-based DCM program that has demonstrated effectiveness with diverse populations in a range of clinical settings. Healthy IDEAS is an evidence-based DCM program that integrates depression awareness and management into existing case management services for older adults. The non-DCM programs included other psychotherapy and case management program models. We collected the following data: PEARLS counselors completed (1) the fidelity instrument at two time points, and (2) a survey about their clinical experience at one time point. Clinical supervisors for the PEARLS programs completed the fidelity instrument on behalf of each counselor that they supervised at one time point. We gave the counselors and supervisors the option of completing the fidelity instrument and clinical experience survey online or with a paper and pencil.

The preliminary analysis involved evaluating mean scores and SDs at the site-level and the individual level using paired *t*-tests. We compared the mean scores for PEARLS sites against the mean scores for the other DCM programs and the non-DCM programs. We also compared site-level mean scores by experience level. We compared individual-level scores by education level (up to 4 years of college and master’s degree), by counselor experience (<1 year, 1–3 years, 4–7 years, and 8 or more years), and by PEARLS-specific experience (<1 year, 1–3 years, and 4 or more years). Instrument validity was assessed using sensitivity and specificity, and we used this information to calculate optimal cut-off scores. We also conducted ROC curve analysis, calculating weighted and unweighted areas under the curve (AUC) as a quantitative method for combining sensitivity and specificity into a single metric. Reliability was evaluated using inter-class coefficients (ICC) between the two survey administration time points.

### Association with client outcomes

For this study, we worked with eight community partners in four states around the U.S. (CA, NY, WA, VT) to examine the relationship between PEARLS program fidelity and PEARLS client outcomes. These PEARLS programs were based on aging, social services, and mental health agencies and represent diverse geographic settings (urban, rural, suburban), and racial/ethnic minority communities (including African-Americans, Filipinos, and other Asian immigrant communities). Many of these programs serve persons with limited income (as indicated by their eligibility for Medicaid and other assistance programs with less than a high school or college education. Each PEARLS program included one to five PEARLS counselors. The four WA PEARLS programs work with one clinical supervisor. The four PEARLS programs outside of Seattle have their own clinical supervisor. Each PEARLS program graduates up to 25 clients over a 6-month period. Agencies were selected based on their current implementation of PEARLS for at least 1 year and their participation on regular PEARLS technical assistance conference calls. We obtained memorandums of understanding from each participating PEARLS program.

We assessed PEARLS program fidelity by administering the PEARLS fidelity instrument to PEARLS counselors and clinical supervisors. PEARLS client outcomes were obtained from existing de-identified PEARLS client outcome data from clients of participating PEARLS counselors. All PEARLS counselors at participating PEARLS programs were invited to participate in this study. Each counselor was asked to complete the fidelity instrument at two time points over the course of the study. In addition, each counselor provided basic information about her or his clinical experience and demographics. No identifiable information was collected about the counselor. We also invited clinical supervisors at each participating agency to complete a PEARLS fidelity instrument on each participating counselor. We linked the PEARLS clinical supervisor fidelity instrument data to the PEARLS counselor data using a unique code assigned for study purposes only. Each clinical supervisor also provided basic information about their clinical experience and demographics. No identifiable information was collected about the clinical supervisor.

We obtained de-identified depression outcome data from the PEARLS clients of each participating PEARLS counselor 6-months following the fidelity survey administration. The outcome data include baseline and final overall and item PHQ-9 depression scores, as had been done in our prior research (2, 4, 5) examining outcomes from treating clients with major depression. We also obtained data on client age, gender, race/ethnicity, and language spoken. We worked with each participating PEARLS agency to ensure that appropriate human subjects protections were in place before obtaining client data. We analyzed the relationship between PEARLS program fidelity total and item scores with the mean change in PHQ-9 scores between participants’ baseline and final PEARLS session. We used Spearman’s rank correlation to measure the degree of association between the PEARLS program fidelity scores and the mean change in PHQ-9. We also dichotomized the total PEARLS fidelity score and looked at whether falling above or below the cut-off predicted significant differences in the mean change in the PHQ-9, using independent *t*-tests to evaluate this difference. Lastly, we summarized responses to the fidelity instrument to examine what adaptations are being made in PEARLS implementation.

## Results

### Instrument development

Seventeen informants provided input on the initial development of the PEARLS fidelity instrument. Ten people participated in the interviews to identify key components: four program developers and researchers, three program administrators, and three PEARLS counselors. Seven former PEARLS clients participated in focus groups to identify the core, programmatic components of PEARLS from their perspective.

The interview and focus group participants identified 42 program components, including items related to training, supervision, treatment, and eligibility. Eighteen items received a high-low score during the Q-sort ranking process, suggesting higher priority for inclusion in the fidelity instrument. After these 18 items were presented back to the interview and focus group participants, two additional items were added, resulting in a total of 20 multiple-choice items. Each item had five possible text anchor points (score of “1” = least fidelity to “5” = highest fidelity), with the total possible score ranging from 20 to 100. The interview participants reviewed the anchor points, and provided feedback. We modified the anchor points based on that feedback.

Questions are divided into two sections: Program Design and Program Delivery. The Design section includes questions about how the organization implements PEARLS, including training, clinical supervision, client recruitment and referrals, and eligibility criteria. The Delivery section focuses on how the counselor implements PEARLS with their clients, such as the average number of sessions that are delivered at home or that identify and discuss social activities. A copy of the resulting PEARLS Fidelity instrument is provided in Data Sheet 1 in Supplementary Material.

### Preliminary evaluation

We used known-groups method data analysis to compare 12 depression programs in six states: six PEARLS programs, four other DCM programs, and two non-DCM programs. Fifty-two PEARLS counselors and seven clinical supervisors provided responses to both the fidelity instrument and the experience survey: 16 respondents from six PEARLS programs, 23 respondents from four DCM programs (one IMPACT program and three Healthy IDEAS programs), and 20 respondents from two non-DCM programs. One practitioner from a DCM site was excluded, due to missing data. Seventy-three percent of the participants completed the fidelity instrument online. It took an average of 14 min to complete. Participants averaged 48 days between the first and second time point for taking the survey.

#### Individual level (PEARLS)

The range of scores was 40–89 for the PEARLS counselors (*n* = 16). Five counselors attended up to 4 years of college and 10 counselors held Master’s degrees. Six practiced as a counselor for <1 year, five practiced for 1–3 years, and five practiced for over 8 years. No counselors practiced from 4–7 years. Seven counselors implemented PEARLS for <1 year, five implemented PEARLS for 1–3 years, and two implemented PEARLS for over 4 years. The sample size was too small to yield significant results on the comparison between education level, experience as a counselor, and experience with implementing the PEARLS program.

#### Site-level (PEARLS, DCM, non-DCM)

**Figure 1:**
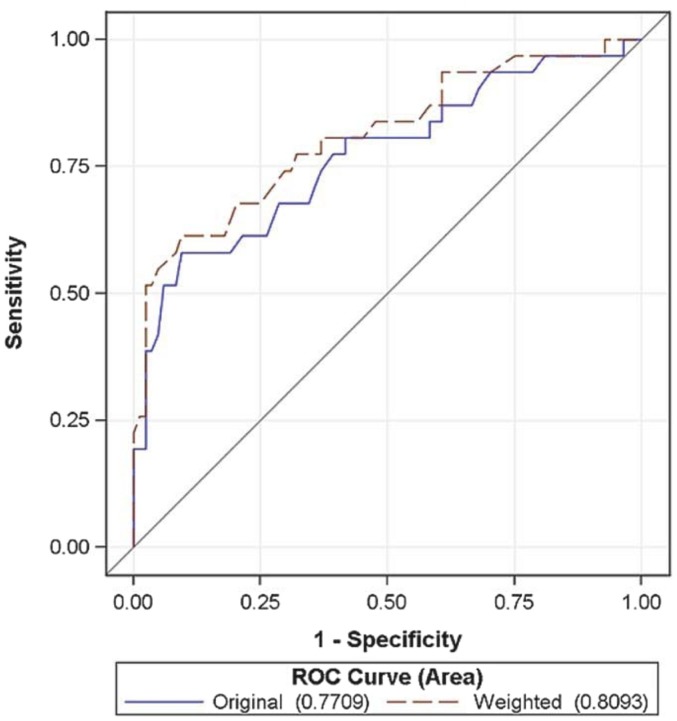
**ROC curves for comparisons**.

Program for Encouraging Active and Rewarding Lives sites reported the highest fidelity score [Mean (SD) 70 (15.5)] compared to sites delivering other DCM programs [55.2 (19.1)] and non-DCM programs [58.0 (13.0)] (*p* < 0.05). Average item scores were 3.56 (0.77) for PEARLS sites compared to 2.9 (0.8) for other DCM sites and non-DCM sites. PEARLS sites with more years of experience reported higher scores (mean 81, range 74–89) than newer programs (mean 59, range 26–81). PEARLS supervisors (*n* = 7) from three PEARLS programs completed the fidelity instrument. Mean fidelity scores were comparable between the supervisor and counselor for all three programs [83 (9) for supervisors and 82 (7) for counselors, *p* = 0.87 (NS)]. Individual item scores were similar, with an average difference of 0.04 between items. Unweighted scoring yielded an AUC in ROC analyses of 0.77. Weighting the overall score improved ROC scores yielding an AUC of 0.81. Optimal cut-off scores for weighted PEARLS fidelity score is 77, yielding a sensitivity of 77% and specificity of 67% for identifying PEARLS counselors and non-PEARLS counselors, respectively (Figure 1). Inter-rater reliability was satisfactory, with an overall ICC of 0.77.

### Association with client outcomes

Twenty-six PEARLS counselors and six clinical supervisors completed the PEARLS fidelity instrument. The mean PEARLS fidelity score was 79.75 (8.33), which was similar to the average total score of 70 (15) for PEARLS programs in the preliminary evaluation. In contrast, the PEARLS counselor and PEARLS clinical supervisor scores differed by an average of 12.17 points (8.09), with some counselors reporting lower scores and others reporting higher scores than their clinical supervisor, even within the same agency.

Program for Encouraging Active and Rewarding Lives participant data were obtained for 127 persons with a mean age of 69 years (8.87). 38.2% identified as White, 26% identified as Hispanic (59% as Mexican), 21.1% as Asian (mainly Filipino with some Vietnames and Korean participants), 11.4% as African-American, and as other races. Only 21% (26 clients) provided data on their income, with all but three of these clients reporting very low income as defined by the Federal Poverty Level, Median Income, or Housing and Urban Development criteria depending on the PEARLS program. Almost half (46.7%) of the respondents that provided information on language spoken at home (*n* = 96) reported speaking a language other than English. Half of those reporting who they lived with stated that they currently lived alone. We did not collect data on education. The mean change in PHQ-9 was 8.79 (5.50). There was little correlation between PEARLS program fidelity and participant outcomes. The correlation between the overall fidelity score and mean change in PHQ-9 was −0.069 (*p* = 0.444). Several fidelity items were significantly correlated with the mean change in the PHQ-9, but all suggested weak correlations. The most strongly correlated items were those that involved the administration of the PHQ-9 (*r* = 0.231, *p* = 0.009), the use of problem-solving treatment (*r* = 0.227, *p* = 0.010), and the use of homework in between in-person sessions (*r* = 0.227, *p* = 0.010).

We conducted additional analyses removing outliers-data for those counselors and clinical supervisor pairings that had a difference in total fidelity score that was 13 or greater (higher than the mean difference between counselors and clinical supervisors of 12.17). Eighty-four PEARLS participants and 16 counselors were included in this revised dataset. The mean change in PHQ-9 was slightly higher than in the original group [9.00 (5.25)]. The mean (SD) total PEARLS fidelity score was also higher [83.42 (5.29)].

We dichotomized the full dataset to look at whether falling above or below a cut-off for the PEARLS fidelity total score predicted a difference in the mean change in PHQ-9 score. Using the cut-off of 70 (as identified in the preliminary evaluation described above), there was not a significant difference between mean change in PHQ-9 between those falling above or at 70 [*n* = 106, 9.16 (5.42)] and those falling below 70 [*n* = 21, 6.90 (5.69), *p* = 0.086]. The difference was also not significant when the cut point was set at 80 (the mean total PEARLS fidelity score in this correlation study), with a mean PHQ-9 change of 8.94 (5.38) (*n* = 81) for those at or above the cut point and 8.52 (5.78) (*n* = 46) for those below the cut point.

We summarized the responses to the fidelity instrument in Table 1. The table provides a snapshot of how PEARLS programs are implementing PEARLS compared to the original research model. Differences exist for clinical supervision, counselor assessment, client eligibility, and the content and format of PEARLS sessions.

**Table 1:** **Research to practice: a summary of responses to the PEARLS fidelity instrument as compared to the original PEARLS model**.

Original PEARLS model	Practice model
**CLINICAL SUPERVISION**	
Formal contract with supervisor	89% have formal supervision in place
Bi-monthly supervision	40% meet at least monthly
Each client discussed at each session	Range from “as needed” (16%) to “weekly” (24%)
**COUNSELOR ASSESSMENT**	
Audiotapes of PEARLS sessions	56% assessed during formal clinical supervision; 28% during job supervision or self-assessment
**ELIGIBILITY**	
Home-based program	42% deliver PEARLS outside of the home
Older adults (60+)	40% include younger adults (<age 60 years)
**PEARLS SESSIONS**	
6–8 in-person sessions	71% average ≥6 sessions per client
PHQ-9	80% PHQ-9 at ≥6 sessions
Education about depression using both written and verbal materials	64% counselors use both written and verbal materials
Sessions focus on the present	42% ≤2 sessions focus on the past
Client chooses problems and solutions	42% ≥6 sessions
Homework completed	17% ≥6 sessions, 75% ≥4 sessions
Behavioral activation	46–58% ≥6 sessions
Written PST worksheet	46% ≥6 sessions

## Discussion

The PEARLS fidelity instrument is a brief, easy-to-use, low-cost option for PEARLS program staff to assess fidelity during program implementation. The tool takes an average of 14 min to complete, allowing for routine assessments by clinical supervisors and counselors. For example, a PEARLS program may use the fidelity instrument periodically as a way to assess whether a counselor is continuing to maintain fidelity to the original program model, and to identify where adaptations have been made. Clinical supervisors may use the instrument to guide ongoing clinical supervision sessions and activities. Funders of PEARLS programs and agency administrators may be interested in the PEARLS fidelity instrument as a quality assurance tool to guide ongoing implementation.

It is surprising that fidelity to the PEARLS program and client outcomes are not more strongly correlated. Also, the discrepancy between clinical supervisor and counselor ratings suggests that there is variation in how each party completes the instrument, and perhaps in how clinical supervisors are involved in the program. We know, for instance, from PEARLS technical assistance activities that some clinical supervisors are more intimately involved with regularly providing supervision that helps guide counselors in adhering closely to the PEARLS model, while others are brought in less frequently and advise more on co-occurring chronic conditions and medication use than on the PEARLS model. More rigorous research is needed to confirm the effectiveness of the tool (e.g., by comparing the tool to the current gold standard of an in-person, full-day, external evaluation of fidelity) before it is disseminated widely.

Research suggests that translation of evidence-based programs must be completed systematically, both at the site-level and individual level, to assure effectiveness (6). The RE-AIM framework is an important tool that is used by researchers and public health practitioners to inform that dissemination. The framework defines translation across five areas – Reach, Effectiveness, Adoption, Implementation, and Maintenance (6). Assuring the fidelity of a particular program to its original evidence-based design is an important aspect of the implementation phase of dissemination (7–14).

It is important to evaluate PEARLS counselors at the site-level to assure that clients are receiving the best possible treatment. It is also imperative to look at the PEARLS program at the site-level to prove to funding agencies that it is effective (6–14). This is particularly important as many funding sources now require some measure of quality assurance or fidelity for supporting evidence-based programs (e.g., Administration on Aging Title III-D funding for evidence-based disease prevention programing). However, many sites lack adequate funding and staff capacity to conduct in-depth, programmatic assessments; therefore, it is important to develop fidelity tools that are effective, user-friendly, and operate at low-cost.

The PEARLS mean score on the fidelity instrument was lower than expected (70.5 out of a possible 100), and Table 1 illustrates some of the changes that agencies implementing PEARLS are making. From our work providing technical assistance to PEARLS sites, we believe that a couple of factors may be at play. Many sites have made programmatic changes to address the needs of their staff and the local population, or due to funding and staffing constraints. For instance, while programs would like to meet weekly with their clinical supervisor, they may meet less frequently due to supervisor availability, limited funds to support the supervisor time, and/or smaller PEARLS client caseloads, which make more frequent supervision unnecessary. Another example is the case of programs delivering PEARLS outside the home. This shift has occurred driven by client preferences to meet at places where they are already congregating (e.g., after a nutrition program at a community center) or preferences to meet elsewhere when a spouse or caregiver is at home due to privacy concerns. Some of the changes are to be expected given that the original PEARLS model was based on the research protocol during an RCT. Elements such as having the clinical supervisor review audiotapes of each PEARLS session are not feasible for community-based agencies that do not have the funds, staffing, or resources. Nor is this level of supervision required for the program to be implemented successfully.

Another example is expanding participant eligibility criteria to better align with the multifaceted, complex clients that agencies see every day. Previous research (21) suggested that the strict PEARLS eligibility criteria was screening out more clients than screening them in, frustrating providers who repeatedly refer clients who are ultimately not treated. During our work with PEARLS providers, we have focused their assessment of client eligibility more on client function, whether they are able to attend PEARLS sessions and to do the work during and in between the sessions. In addition, we know that PEARLS programs are making adaptations to fit their local populations. Sites do not use written information with every participant as some are illiterate or have low literacy, or speak a language other than English and materials are interpreted versus translated. In addition, some sites allow younger older adult participants in the program (typically age 50–59 years) as they have no other treatment options, are seen in similar settings, and are as successful as the 60+ population.

These findings also point to the fact that fidelity is only a piece of the implementation of PEARLS. The weak, generally non-significant correlations between fidelity score and client outcomes may suggest that other factors are at play, which impact whether a client improves their depressive symptoms. From our work providing technical assistance to PEARLS programs, we know that at the individual level, motivation, stigma about depression, physical health and management of other comorbidities, informal and formal support, and mobility and function are all factors that influence whether someone successfully participates in PEARLS. At the site-level, other approaches for improving EBP implementation include outcome monitoring, regular and structured supervision, effective organization and climate, rigorous selection and retention of team members, and ongoing consultation and technical assistance (21).

After measuring fidelity, it is important to then provide fidelity feedback to EBP providing agencies so that they can modify their services based on feedback from their fidelity reviews (22). One example of a systematic approach for providing fidelity feedback is from the National Implementing Evidence-Based Practices Project (23). Most of the successfully implementing sites had altered their services based on feedback received during their fidelity reviews, suggesting that the fidelity review process can be effective (22). With PEARLS, we have shared findings from each phase of this fidelity study with participants on our monthly conference calls to provide technical assistance.

The fidelity instrument that we developed in this study may be the most effective way to evaluate effective implementation in various locations and community settings across the country. While independent, observational measures of fidelity are ideal, this instrument provides a practical, user-friendly tool that programs can use internally and at minimal cost for monitoring program quality. Further work is necessary to ascertain the validity of the instrument given the discrepancy between counselor and supervisor ratings and it may need refinement to correlate more strongly with client outcomes.

## Conflict of Interest Statement

The authors declare that the research was conducted in the absence of any commercial or financial relationships that could be construed as a potential conflict of interest.

This paper is included in the Research Topic, “Evidence-Based Programming for Older Adults.” This Research Topic received partial funding from multiple government and private organizations/agencies; however, the views, findings, and conclusions in these articles are those of the authors and do not necessarily represent the official position of these organizations/agencies. All papers published in the Research Topic received peer review from members of the Frontiers in Public Health (Public Health Education and Promotion section) panel of Review Editors. Because this Research Topic represents work closely associated with a nationwide evidence-based movement in the US, many of the authors and/or Review Editors may have worked together previously in some fashion. Review Editors were purposively selected based on their expertise with evaluation and/or evidence-based programming for older adults. Review Editors were independent of named authors on any given article published in this volume.

## Supplementary Material

The Supplementar Material for this article can be found online at http://www.frontiersin.org/Journal/10.3389/fpubh.2014.00200/abstract
