# Supplementary material for: Development and Evaluation of a Fidelity Instrument for PEARLS
Source: Front Public Health. 2015 Apr 27;2:200. doi: 10.3389/fpubh.2014.00200 (PMC4410416; doi:10.3389/fpubh.2014.00200)
Supplement: Supplementary file 1 [file Data_Sheet_1.PDF]

UNIVERSITY OF WASHINGTON PEARLS FIDELITY STUDY

START TIME: ______________________

PEARLS FIDELITY INSTRUMENT

Please select the response (1 through 5) that best fits the depression treatment that you deliver.

PROGRAM DESIGN

1. Your training in depression treatment was done via:

☐ (1) Written materials only (e.g. manual)
☐ (2) Media- based training (e.g. DVD, web)
☐ (3) In person, not including Problem Solving Treatment (PST)
☐ (4) In person, including practice sessions and PST, non-University of Washington (UW) Sponsored
☐ (5) In person, including practice sessions and PST, UW sponsored

2. Your ability to adequately provide treatment was evaluated via:

☐ (1) During job supervision (by non-clinical supervisor)
☐ (2) Self-assessment
☐ (3) During in person clinical supervision
☐ (4) Direct observation of treatment sessions by clinical supervisor
☐ (5) Audiotape assessment by clinical supervisor

3. People making referrals receive education to increase awareness of late life depression, the treatment, and the referral process. This education is done via:

(Score the highest point for the approach that you routinely use)
☐ (1) Information provided as part of other staff trainings
☐ (2) Information posted on bulletin board (not individualized)
☐ (3) Individualized written information
☐ (4) In-person presentation from someone not part of the treatment team
☐ (5) In-person presentation from treatment team member

4. Your clinical supervision is provided via:

☐ (1) Arrangement with non-clinical supervisor
☐ (2) Informal arrangement with clinical supervisor
☐ (3) Routine peer supervisor
☐ (4) Formal arrangement with other clinical supervisor
☐ (5) Formal arrangement with psychiatrist

5. Supervision of your depression treatment occurs:

PILOT TOOL

☐ (1) In-person or phone, less than monthly, unplanned meetings
☐ (2) In-person or phone, at least monthly (less often than two times per month) planned meetings
☐ (3) In-person or phone, at least two times per month, unplanned meetings
☐ (4) In-person or phone, at least two times per month, planned meetings
☐ (5) In-person or phone, weekly, planned meetings

6. A typical client case is reviewed in supervision:

☐ (1) On "as needed" basis
☐ (2) Every 2-3 months
☐ (3) Monthly
☐ (4) Two times a month
☐ (5) Weekly

Please choose all items that apply for question number 7.

7. Treatment inclusion criteria include:

☐ Living independently at home
☐ Not cognitively impaired
☐ Not functionally impaired by other psychiatric diagnoses (e. g. bipolar)
☐ Diagnosis/PHQ-9 screening outcome of minor depression
☐ No one under 60 years of age

PROGRAM DELIVERY

8. Participant education about depression symptoms and treatment approach is provided through:

☐ (1) Written information about depression
☐ (2) Verbal information about depression
☐ (3) Written OR verbal information about the intervention
☐ (4) Verbal OR written information about depression and the intervention
☐ (5) Written and verbal information about depression and the intervention

Go to page 3 for questions #9 - #20.

PILOT TOOL

For items #9-20, consider the average number of sessions <u>per participant, over the last 6 months</u>

	Average of 2 or less sessions	Average of 3 sessions	Average of 4 sessions	Average of 5 sessions	Average of 6 or more sessions
9. Average number of in-person sessions:	☐	☐	☐	☐	☐
10. Average number of sessions where a validated depression scale (e.g. PHQ-9) is completed:	☐	☐	☐	☐	☐
11. Average number of sessions where the treatment is used:	☐	☐	☐	☐	☐
12. Average number of sessions in which problem solving treatment includes written problem and solutions strategies:	☐	☐	☐	☐	☐
13. Average number of sessions where physical activities/exercise is identified and discussed:	☐	☐	☐	☐	☐
14. Average number of sessions where social activities are identified and discussed:	☐	☐	☐	☐	☐
15. Average number of sessions where pleasant events are identified and discussed:	☐	☐	☐	☐	☐
16. Average number of treatment sessions in which homework is completed:	☐	☐	☐	☐	☐
17. Average number of treatment sessions delivered in the client's home:	☐	☐	☐	☐	☐
18. Average number of sessions in which problems and solution strategies are chosen by the participant:	☐	☐	☐	☐	☐
19. Average number of sessions in which the treatment focuses on the participants past:	☐	☐	☐	☐	☐
20. Average number of sessions where the focus is centered around behavioral change:	☐	☐	☐	☐	☐

PILOT TOOL

STOP TIME: ________________________

Thank you for completing this survey!

PILOT TOOL
